# Activation of TLR7-mediated autophagy increases epileptic susceptibility via reduced KIF5A-dependent GABA_A_ receptor transport in a murine model

**DOI:** 10.1038/s12276-023-01000-5

**Published:** 2023-06-01

**Authors:** Jing Liu, Pingyang Ke, Haokun Guo, Juan Gu, Yan Liu, Xin Tian, Xuefeng Wang, Fei Xiao

**Affiliations:** 1 Department of Neurology, The First Affiliated Hospital of Chongqing Medical University, Chongqing Key Laboratory of Neurology, 1 Youyi Road, Chongqing, 400016 China; 2 Department of Neurology, Chongqing University Three Gorges Hospital, 165 Xincheng Road, Chongqing, 404100 China; 3 Institute for Brain Science and Disease of Chongqing Medical University, Chongqing, 400016 China

**Keywords:** Epilepsy, Synaptic transmission

## Abstract

The pathophysiological mechanisms underlying epileptogenesis are poorly understood but are considered to actively involve an imbalance between excitatory and inhibitory synaptic transmission. Excessive activation of autophagy, a cellular pathway that leads to the removal of proteins, is known to aggravate the disease. Toll-like receptor (TLR) 7 is an innate immune receptor that regulates autophagy in infectious and noninfectious diseases. However, the relationship between TLR7, autophagy, and synaptic transmission during epileptogenesis remains unclear. We found that TLR7 was activated in neurons in the early stage of epileptogenesis. TLR7 knockout significantly suppressed seizure susceptibility and neuronal excitability. Furthermore, activation of TLR7 induced autophagy and decreased the expression of kinesin family member 5 A (KIF5A), which influenced interactions with γ-aminobutyric acid type A receptor (GABA_A_R)-associated protein and GABA_A_Rβ2/3, thus producing abnormal GABA_A_R-mediated postsynaptic transmission. Our results indicated that TLR7 is an important factor in regulating epileptogenesis, suggesting a possible therapeutic target for epilepsy.

## Epilepsy: a receptor protein that can take a toll

Studies using a mouse model of epilepsy implicate a protein inside cells called Toll-like receptor 7 (TLR7) in susceptibility to epileptic seizures, suggesting the protein could be a target for antiepileptic drugs. TLR7 is part of the immune system and is found in the external and internal membranes of some cells, recognizing molecules associated with infection and disease. It can, however, lead to damaging over-reactions, including auto-immune responses. Fei Xiao, Xuefeng Wang, and colleagues at Chongqing Medical University in China detected activation of TLR7 in mouse neurons during the cellular changes that underly a susceptibility to epilepsy. They also identified molecular signaling pathways allowing TLR7 activity to disturb nerve transmission by modulating processes that normally degrade used cell components. TLR7 and related proteins may also be involved in other noninfectious diseases of the central nervous system.

## Introduction

Epilepsy is a serious and highly disabling neurological disease that affects 7.6‰ of people of all ages worldwide and is characterized by highly synchronized abnormal firing of massive neurons^1^. Although more than 20 antiseizure drugs have been introduced over the past 30 years, no antiepileptic medicines can prevent epileptogenesis^2^, which is the continuous and prolonged process that precedes spontaneous seizures. Disordered molecular and cellular plasticity during epileptogenesis are believed to be the reason for the first unprovoked seizure(s), which may persist indefinitely afterward, thus contributing to the progression of epileptic conditions^3^. Temporal lobe epilepsy (TLE) is one such progressive disease^4^.

Toll-like receptors (TLRs) are involved in recognition of pathogen-associated molecular patterns (PAMPs) and induce innate immune responses through pattern recognition receptors. TLRs play a major role in both infectious and noninfectious central nervous system (CNS) diseases in which pathogen-associated molecules are undetectable. Notably, TLR7 is mainly expressed in neurons of the CNS and microglia, the major immune cells of the brain. TLR7 reportedly participates in noninfectious CNS diseases, such as Alzheimer’s disease (AD)^5^, Parkinson’s disease (PD)^6^, and stroke^7^. Although TLR7 is reportedly activated during epilepsy associated with tuberous sclerosis complex^8^, the role of TLR7 in TLE, whose prominent feature is neuroinflammation^9^, has not been reported.

Macroautophagy, also known as autophagy, is a major degradation pathway through which unwanted protein substances in autophagosomes accumulate and are degraded by lysosomes^10^. Excessive autophagy has been implicated in severe stress and shown to exacerbate disease^11–14^. Autophagic activity can lead to cell survival during mild seizures (Racine stage 1–2), whereas dysregulated autophagy results in the polarization of microglia to a proinflammatory M1 phenotype during severe seizures (Racine stage ≥3) and may play a role in the initiation and exacerbation of epilepsy^15^. Nevertheless, the role of hippocampal autophagy in the epileptogenesis of TLE remains unclear.

The mechanism of epilepsy is based on hyperexcitability, which is attributed to an imbalance between neuronal excitation and inhibition^16^. γ-Aminobutyric acid (GABA) is the major inhibitory neurotransmitter in the CNS and acts through GABA type A (GABA_A_) and type B (GABA_B_) receptors. GABA_A_ receptors (GABA_A_Rs) are primary inhibitory neurotransmitter-gated ion channels in the mammalian CNS^17^. Dysfunction leading to increased synaptic excitation and/or reduced inhibition results in epilepsy.

Therefore, the aim of this study was to investigate the role of TLR7 in epileptogenesis using a mouse model of TLE by exploring (1) the expression and distribution of TLR7 in the hippocampus during the period of epileptogenesis; (2) the capacity of TLR7 to regulate neuronal excitability and seizure susceptibility; and (3) the disruptive effects of TLR7 on balance between neuronal excitation and inhibition via autophagy.

## Materials and methods

### Animals

*Tlr*7^−/−^ and *Tlr7*^−/y^ mice (*Tlr*7^tm1Flv^/J, RRID: IMSR_JAX:008380) on a C57BL/6 genetic background were imported from the Jackson Laboratory (Bar Harbor, ME, USA). Male C57BL/6 mice were purchased from the Laboratory Animal Centre of Chongqing Medical University (Chongqing, China). All mice were kept and bred under specific-pathogen-free conditions with a 12-h light/dark cycle and controlled temperature and humidity. The *Tlr7* gene is located on the X chromosome. *Tlr7*^−/y^ represents male mice used in this study. Sex was not distinguished for neonatal mice from which the primary hippocampal neuronal cultures were generated. *Tlr7*^−/−^ indicates *Tlr7* knockout in neurons. All animal experiments were approved by the Animal Experimental Ethics Committee of Chongqing Medical University and conducted in accordance with the Guidelines for the Care and Use of Laboratory Animals issued by the National Health Research Institute.

### TLE model and electroencephalogram recordings

#### Establishing the TLE model

Intrahippocampal administration of kainic acid (KA) has been extensively described for establishing models of TLE^18^. Adult male mice weighing 26–30 g were anesthetized by intraperitoneal (i.p.) injection of pentobarbital (100 mg/kg), mounted on a stereotaxic apparatus, and injected with 1.0 nmol KA (Sigma‒Aldrich, St. Louis, MO, USA) in 50 nL saline (KA group) or with 50 nL saline (control group). Injections were administered in the dorsal hippocampal region using the following coordinates from bregma: anteroposterior, −2 mm; mediolateral, −1.5 mm; dorsoventral, −1.5 mm. After injection, the syringe was maintained in position for an additional 30–60 s to avoid backflow. Mice were returned to their cages, and their seizure development status was monitored. Seizures were rated using the Racine scale (for details, see Supplementary Material)^19^. Only mice with seizures above Racine stage three were selected for subsequent experiments.

#### Electrode implantation

Mice were anesthetized with pentobarbital (100 mg/kg, i.p.), mounted on a stereotaxic apparatus, and electrodes were implanted in the brain cortex using a fixed head-mounted device (for details, see Supplementary Material). Five days after recovery, mice were subjected to i.p. injections of KA.

#### Seizure induction via intraperitoneal injection of KA

I.P. administration of KA has been widely used to study seizure susceptibility in TLE models. Although spontaneous recurrent seizures (SRSs) are rarely observed, the use of KA via i.p. injection can avoid the effects of anesthetics. The dose of KA used to induce status epilepticus (SE) was 20 mg/kg for adult mice^20^. To assess seizure susceptibility, seizures were rated using the Racine scale.

#### Electroencephalogram (EEG) recordings and data analysis

EEG recordings were performed as previously described in ref. ^21^. Briefly, the head mount was connected to the preamplifier, followed by acclimation for 15 min. A baseline recording was obtained for 5 min using a three-channel tethered EEG system (Pinnacle Technology, Lawrence, KS, USA) with Sirenia Acquisition software v2.2.1 (Pinnacle Technology), employing a 2 kHz sampling rate, 0.5 Hz high-pass filter, and 100 Hz low-pass filter. I.P. injections of KA were used to induce SE, and EEG recordings were conducted continuously for three days afterward. During the EEG recordings, mice moved freely and consumed an experimental diet. Seizures in EEG were recognized on characteristic spike-wave EEG discharges.

### Brain tissue samples

For western blotting, RNA, and coimmunoprecipitation analyses, mice were anesthetized with pentobarbital (100 mg/kg, i.p.) on the third day after intrahippocampal injection of KA. Cortical and hippocampal tissues were obtained on ice. Brain tissue samples were either immediately used or frozen in liquid nitrogen until further use.

For immunofluorescence analysis, mice were anesthetized with pentobarbital (100 mg/kg, i.p.) on the 3rd day after intrahippocampal injection and transcardially perfused with 30 mL ice-cold 1× phosphate-buffered saline (PBS) followed by 30 mL ice-cold 4% paraformaldehyde (PFA) in 1×PBS. The excised brain was fixed for 24 h in PFA at 4 °C and then cryoprotected in 20% sucrose for 18 h and in 30% sucrose for 36 h before sectioning. The brain tissues were surrounded by an optimal cutting temperature compound, frozen in dry ice-chilled isopentane, and sliced into 10-mm coronal sections using a Leica cryostat (Wetzlar, Germany). The sectioned brain tissues were stored at −80 °C until immunofluorescence analysis.

### Reverse transcriptase quantitative PCR (RT‒qPCR)

Total RNA was isolated from cortical and hippocampal tissues using TRIzol reagent in accordance with the manufacturer’s protocol (Invitrogen, Carlsbad, CA, USA). Total RNA concentrations and purity were measured using a Nanodrop 2000, and cDNA was synthesized using HiScript II Q Select RT SuperMix for qPCR (Vazyme Biotech, Nanjing, China) following the manufacturer’s instructions. RNA (600 ng) was used to synthesize cDNA in a total volume of 20 μL. cDNA template (1 μL) was employed for real-time PCR using the SYBR Green qPCR Master Mix Kit (Vazyme Biotech) with the StepOnePlus Real-Time PCR system (Applied Biosystems, Foster City, CA, USA). *Gapdh* was used as a reference gene. Each sample was evaluated in triplicate or in duplicate, and gene expression was expressed as fold changes by the comparative Ct method (2^−ΔΔCt^). The primer sequences are shown in Supplementary Table 1.

### Western blot and immunofluorescence analysis

Western blot and immunofluorescence analysis of hippocampal and cortical tissues was performed as described in the Supplementary Material. The following primary antibodies were used: for western blot analysis, TLR7 (1:500; Cat. NBP2-24906, RRID: AB_2922764; Novus Biologicals, Littleton, CO, USA), Beclin1 (1:200; Cat. sc-48341, RRID: AB_626745; Santa Cruz Biotechnology, Dallas, TX, USA), LC3B (1:300; Cat. 18725-1-AP, RRID: AB_2137745; Proteintech, Chicago, IL, USA), P62 (1:1000; Cat. 18420-1-AP, RRID: AB_10694431; Proteintech), KIF5A (1:2000; Cat. 21186-1-AP, RRID: AB_10733125; Proteintech), KIF5B (1:2000; Cat. 21632-1-AP, RRID: AB_11182931; Proteintech), KIF5C (1:2000; Cat. 25897-1-AP, RRID: AB_2880288; Proteintech), GABA_A_R β2/3 (1:500; Cat. bs-12066R, RRID: AB_2922761; Bioss Antibodies, Woburn, MA, USA), GLUR2/3 (1:1000; Cat. A2754, RRID: AB_2922763; ABclonal Technology, Woburn, MA, USA), GAPDH (1:5000; Cat. 10494-AP-1, RRID: AB_2263076; Proteintech), and ATP1A1 (1:5000; Cat. 14418-1-AP, RRID: AB_2227873; Proteintech) were used. For immunofluorescence analysis, TLR7 (1:200; Cat. NBP2-24906, RRID: AB_2922764; Novus Biologicals), NeuN (1:200; Cat. MAB377, RRID: AB_2298772; Millipore, Billerica, MA, USA), IBA1 (1:300; Cat. ab5067, RRID: AB_732306; Abcam, Cambridge, UK), GFAP (1:200; Cat. 16825-1-AP, RRID: AB_2109646; Proteintech), and LC3B (1:100; Cat. 18725-1-AP, RRID: AB_2137745; Proteintech) were used.

### Transmission electron microscopy (TEM)

Mice were anesthetized with pentobarbital (100 mg/kg, i.p.) and subjected to intracardial perfusion with ice-cold 1×PBS followed by 2.5% glutaraldehyde. The brain was removed, and the CA1 region of the hippocampus was cut into small specimens (thickness <1 mm), which were stored in 2.5% glutaraldehyde overnight at 4 °C. The brain tissue samples were sent to the TEM laboratory of Chongqing Medical University for subsequent experimental preparation (for details, see Supplementary Material). The prepared brain sections were observed under an HT7700 transmission electron microscope (Hitachi, Tokyo, Japan).

### Hippocampal neuronal culture and drug administration

Hippocampal neurons were isolated from newborn C57BL/6 mice (P0-P1) and cultured as previously described (for details, see Supplementary Material)^22^. To determine the effect of TLR7-independent autophagic degradation on KIF5A, cultured neurons were treated with either 2 µg/mL imiquimod (IMQ; InvivoGen, San Diego, CA, USA), 10 mM 3-methyladenine (3-MA; MedChemExpress, Monmouth Junction, NJ, USA), 20 nM rapamycin (MedChemExpress), or 0.1% DMSO for 24 h before the neurons were lysed.

### Whole-cell patch-clamp recordings

Mice were deeply anesthetized with pentobarbital (100 mg/kg, i.p.) and decapitated. The brain was quickly and gently removed from the skull and placed in an ice-cold cutting solution. Coronal slices (300 mm) were cut using a VP1200S Vibratome (Leica) and then placed in an interface-type chamber that was continuously oxygenated with artificial cerebrospinal fluid (ACSF). After recovery for at least 1 h, the slices were prepared for recording. Hippocampal CA1 pyramidal cells were visualized using an inverted phase-contrast microscope, and whole-cell recordings were performed using borosilicate glass electrodes (4–6 MΩ).

Current-clamp recordings were conducted to measure spontaneous action potentials (sAPs) at the resting membrane potential. Voltage-clamp recordings were performed at a holding potential of −70 mV, and spontaneous excitatory postsynaptic currents (sEPSCs), spontaneous inhibitory postsynaptic currents (sIPSCs), miniature excitatory postsynaptic currents (mEPSCs) and miniature inhibitory postsynaptic currents (mIPSCs) were recorded. For evoked IPSC (eIPSC) recordings, a bipolar tungsten stimulating electrode was placed in the Schaffer collateral (SC)-CA1 pathway (stimulation rate = 0.05 Hz), and data were recorded at a holding potential of 0 mV. For paired-pulse ratio (PPR) recordings, IPSCs were evoked by paired-pulse stimulation of the SC-CA1 pathway at intervals of 50 ms (stimulation rate 0.1 Hz, 100 mA, and 100 ms duration) and a holding potential of 0 mV. PPR was defined as the ratio between the first and the second evoked amplitude (for details, see Supplementary Material).

All patch-clamp data were obtained using a MultiClamp 700 B patch-clamp amplifier (Axon Instruments, Burlingame, CA, USA) with a 2 kHz low-pass filter and 10 kHz sample rate and were recorded using pClamp 10 software (Molecular Devices, Sunnyvale, CA, USA). The data were analyzed using Clampfit software (Molecular Devices).

### Coimmunoprecipitation

The coimmunoprecipitation and quantitative coimmunoprecipitation assays were performed as previously described in ref. ^22^. Hippocampal tissues from KA-induced epileptic mice were incubated overnight at 4 °C with the following antibodies: KIF5A (1:250; Cat. 21186-1-AP, RRID: AB_10733125; Proteintech), anti-GABA_A_Rβ2/3 (1:500; Cat. bs-12066R; RRID: AB_2922761; Bioss Antibodies), anti-GABARAP (1:450; Cat. 18723-1-AP, RRID: AB_10603365; Proteintech), or IgG (rabbit, Cell Signaling Technology). Subsequently, the mixtures were incubated with protein A/G agarose beads (MedChem Express) overnight at 4 °C. The immunoprecipitated compounds were immunoblotted against the antibodies listed above.

### Intrahippocampal injection of the AAV vector

Due to high KIF5A expression in *Tlr7*^−/y^ mice on the third day after intrahippocampal administration of KA, an shRNA was generated with the targeting sequence 5′-ATGAAGGACAAGCGTAGATAC-3′ directed against *Kif5a*^23^. Designated AAV-*Kif5a*, the shRNA was carried by an AAV to decrease hippocampal KIF5A levels in *Tlr7*^−/y^ mice after intrahippocampal administration. The AAV vector also contained a separate transcription cassette for enhanced green fluorescent protein (EGFP). The control vector Con-shRNA contained an shRNA with the targeting sequence 5′-GAAGTCGTGAGAAGTAGAA-3′.

Subsequently, mice were anesthetized with pentobarbital (100 mg/kg, i.p.) and fixed in a stereotaxic apparatus. Vector particles (1 × 10^12^ TU/mL, 2 μL) were injected into the bilateral dorsal hippocampus using a glass microsyringe (0.2 μL/min). The pipette was maintained in position for 5 min to prevent backflow. After one month, mice in the AAV-*Kif5a*- and Con-shRNA-treated groups were subjected to intrahippocampal or i.p. injection of KA to perform the rescue experiments.

### Statistical analysis

Statistical analysis was performed using GraphPad Prism v 9.0 software (GraphPad Software, La Jolla, CA, USA). Differences between the two groups were assessed using an unpaired two-tailed Student’s *t*-test. Differences between one variable from more than two groups were assessed using one-way ANOVA followed by Tukey’s post hoc test. Differences between two variables from more than two groups were analyzed using two-way ANOVA with the Bonferroni post hoc test. Percent survival was judged by the Mantel‒Cox log-rank test. The sample size was chosen according to that used for similar experiments in the literature. Data were represented as the mean ± SEM. Significant differences were set at *p < 0.05, **p < 0.01, ***p < 0.001, ****p < 0.0001. Nonsignificant differences are indicated by “ns”.

## Results

### TLR7 expression is increased in the KA-induced epilepsy model

**Fig. 1:**
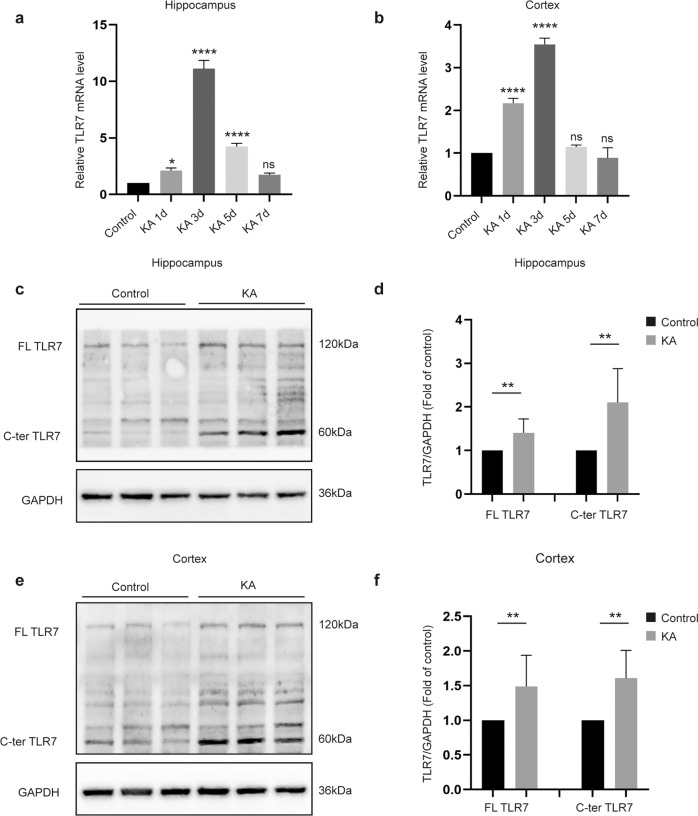
Expression of TLR7 in brain tissues at different time points after induction of status epilepticus. Quantitative real-time PCR analysis of the expression of TLR7 mRNA in the hippocampus (**a**) and cortex (**b**) at different time points after induction of SE (*n* = 3). ns not significant; *P < 0.05; ****P < 0.0001; one-way ANOVA with Tukey’s post hoc test. Bars represent the mean ± SEM. The expression of FL-TLR7 and C-terminal TLR7 in the hippocampus (**c**, **d**) and temporal cortex (**e**, **f**) on the third day after induction of SE by intrahippocampal injection with KA were both significantly higher than that of the control group (*n* = 9 mice per group). **P < 0.01; unpaired two-tailed Student’s *t*-test. Bars represent the mean ± SEM.

The present study determined TLR7 mRNA expression during different stages of epilepsy. TLR7 mRNA expression in the epileptic hippocampus and cortex increased during the latent period, also known as epileptogenesis, following the initial precipitating injury until the appearance of recurrent seizures^19^, and then expression gradually decreased. Changes in TLR7 mRNA expression did not differ significantly between the KA and control groups until the chronic stage (Supplementary Fig. 1a, b). However, TLR7 mRNA expression peaked on the third day after inducing SE with KA (Fig. 1a, b), hereafter named the early stage of epileptogenesis. Western blotting was performed to confirm TLR7 protein levels on the third day after induction of SE. Consistent with the mRNA results, the protein expression levels of full-length (FL, ~120 kDa) and C-terminal (C-ter, ~60 kDa) TLR7 in the hippocampus and cortex of the KA group were significantly higher than those in the control group (Fig. 1c–f).

### KA activates TLR7 mostly in neurons

**Fig. 2:**
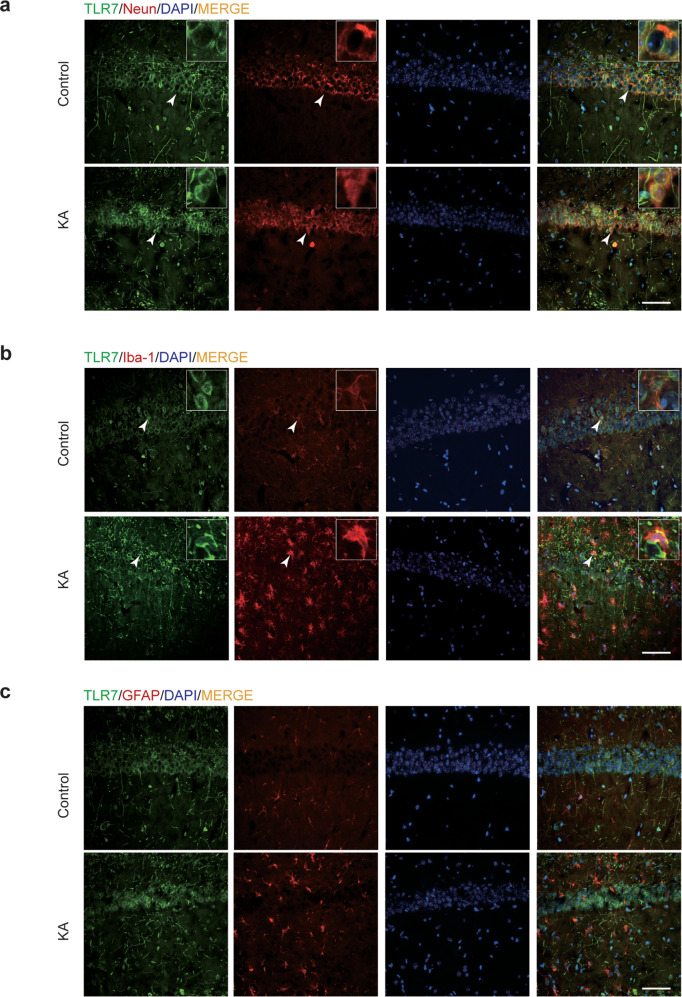
Cellular localization of TLR7 in the CA1 region from control and KA-injected (3 days postinjection) mice. Double immunofluorescence staining showed that TLR7 was activated in KA, and most TLR7 colocalized with NeuN (**a**) and less colocalized with Iba-1 (**b**) but did not colocalize with GFAP (**c**). Arrowheads indicate TLR7-positive cells, NeuN-positive neurons, Iba-1-positive microglia, GFAP-positive astrocytes, TLR7-positive/NeuN-positive neurons, and TLR7-positive/Iba-1-positive microglia. Scale bar = 50 μm.

Cellular localization of TLR7 in the KA-induced epileptic hippocampus has never been reported. Therefore, immunofluorescence staining was employed to reveal the cellular localization of TLR7 in brain tissues on the third day after induction of SE compared to that in control brain tissues. TLR7 predominantly colocalized with neuron-specific nuclear protein (NeuN; a neuronal marker), and enhanced fluorescence intensity was observed in the KA group (Fig. 2a). Some TLR7 colocalized with ionized calcium-binding adapter molecule 1 (Iba-1, a microglia marker) and was accompanied by notably activated microglia in the KA group (Fig. 2b). TLR7 did not colocalize with glial fibrillary acidic protein (GFAP; an astrocyte marker) (Fig. 2c) in the hippocampal CA1 region in either group. Notably, this region undergoes particularly strong and typical changes during epileptogenesis^24^. This specific distribution suggested that TLR7 exerted a regulatory effect mainly through neurons rather than through glia. Furthermore, a similar result was observed in the temporal cortex and hippocampal CA3 region, where TLR7 was activated after KA injection and colocalized with NeuN (Supplementary Fig. 1d).

### TLR7 knockout suppresses seizure susceptibility

**Fig. 3:**
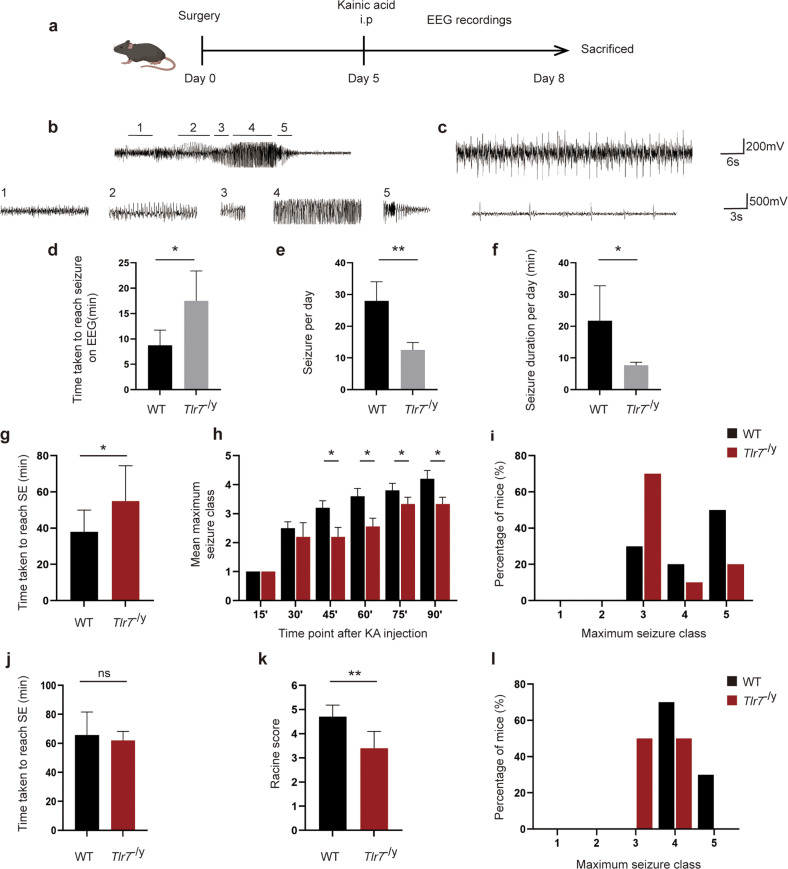
Knockout of TLR7 inhibits seizure susceptibility in KA-injected mice. **a** Simultaneous recording of neuronal activity via EEG/EMG implants. **b** Representative EEG trace showing stages used to define seizures quantified in (1) low-frequency background, with low-voltage spiking, (2) synchronized high-frequency and high-voltage spiking, (3) high-frequency and low-voltage spiking, (4) unsynchronized high-frequency and high-voltage spiking, and (5) high-frequency, with burst spiking. **c** Representative EEG trace showing single spike-wave discharges in the *Tlr7*^−/y^ group. The latency period of seizures (**d**), the average number of seizures per day (**e**), and total duration of seizures per day (**f**) in WT and *Tlr7*^−/y^ mice treated with KA (*n* = 4). **g** Graph depicting the time taken to reach status epilepticus after KA intraperitoneal administration from WT group and *Tlr7*^−/y^ group mice (*n* = 10). **h** Graph depicting seizure progression in WT and *Tlr7*^−/y^ mice, illustrated as the mean maximum seizure class reached by 15, 30, 45, 60, 75, and 90 min after KA intraperitoneal administration (*n* = 10). **i** Incidence of maximum seizure class reached during the course of the experiments in **h** (*n* = 10). **j** Graph depicting the time taken to reach status epilepticus after KA intrahippocampal administration from WT group and *Tlr7*^−/y^ group mice (*n* = 10). **k** Graph depicting the severity of seizures in WT and *Tlr7*^−/y^ mice after KA intrahippocampal administration, illustrated as the mean Racine score (*n* = 10). **l** Incidence of maximum seizure class reached during the course of the experiments in k (*n* = 10). ns, not significant; *P < 0.05; **P < 0.01; unpaired two-tailed Student’s *t*-test. Bars represent the mean ± SEM.

To assess the pathophysiological consequences of TLR7 in vivo, the effect of TLR7 knockout on epileptogenesis was assessed in *Tlr7*^−/y^ mice. First, TLR7 mRNA expression was measured in the WT and *Tlr*7^−/y^ groups using qPCR to verify the efficiency of TLR7 knockout. TLR7 mRNA was not detected in the *Tlr7*^−/y^ group (Supplementary Fig. 1c). Subsequently, mice in both groups were administered intrahippocampal and i.p. injections of KA for further behavioral tests. Epileptic seizures were monitored continuously for three days after KA intraperitoneal injection by EEG (Fig. 3a). The latent period before seizures, as well as seizure frequency and duration, were recorded by EEG. The *Tlr*7^−/y^ group displayed more single spike-wave discharges (Fig. 3c) than epileptiform spike discharges (Fig. 3b). Nonetheless, the *Tlr*7^−/y^ group exhibited an increased latent period and decreased seizure incidence and duration compared to the WT group (Fig. 3d–f).

Further behavioral experiments were then performed using i.p. and intrahippocampal injections of KA. The *Tlr7*^−/y^ group displayed a significantly prolonged latent period after i.p. administration of KA compared to the WT group (Fig. 3g). Moreover, the Racine score and maximum seizure severity were considerably lower in the *Tlr7*^−/y^ group than in the WT group, with fewer mice developing tonic hindlimb extension (Fig. 3h, i). Additionally, the *Tlr7*^−/y^ group showed less seizure progression, which was assessed by measuring the maximal seizure class of the mice every 15 minutes (Fig. 3h). A similar conclusion was reached through intrahippocampal injections of KA. The latent period did not differ significantly between the *Tlr7*^−/y^ and WT groups (Fig. 3j), but the *Tlr7*^−/y^ group exhibited a lower Racine score (Fig. 3k) and maximum seizure severity (Fig. 3l).

Changes in SRSs during the chronic period in TLE model epileptic mice were investigated to determine whether TLR7 knockout during the latent period had an effect on chronic behavior. Mice were continuously monitored by video for one month after the onset of SE. Fewer SRSs and longer latency periods were recorded in the *Tlr7*^−/y^ group than in the WT group (Supplementary Fig. 2a, b). Taken together, the behavioral results indicated that the deletion of TLR7 suppressed seizure susceptibility and epileptic processes.

### TLR7 induces hippocampal neuronal autophagy in the early stage of epileptogenesis

**Fig. 4:**
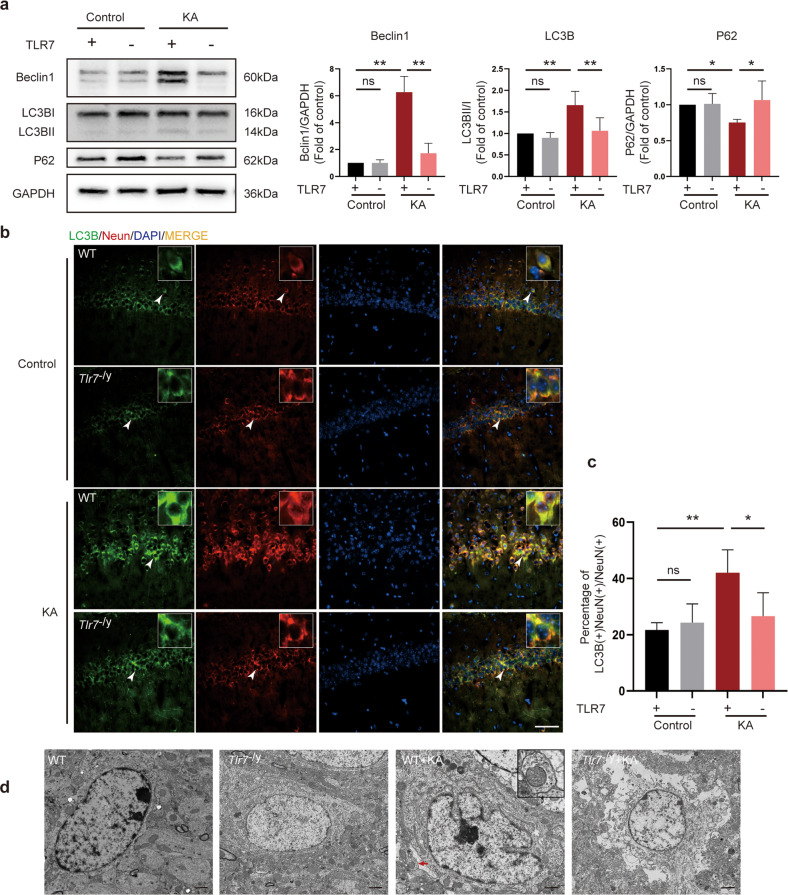
Knockout of TLR7 suppressed neuronal autophagy in KA-injected mice. **a** Representative images of autophagy-related protein (Beclin1, LC3B, and P62) expression were detected by western blot (*n* = 6). Quantitative analysis was performed. ns not significant; *P < 0.05; **P < 0.01; one-way ANOVA with Tukey’s post hoc test. Bars represent the mean ± SEM. **b** Representative images of neurons labeled with NeuN and LC3B in the hippocampal CA1 region. Arrowheads indicate LC3B-positive cells, NeuN-positive neurons, and LC3B-positive/NeuN-positive neurons. Scale bar = 50 μm. **c** Quantitative analysis was performed as the percentage of NeuN and LC3B double-positive cells in NeuN-positive neurons in the hippocampal CA1 region (*n* = 5). ns not significant; *P < 0.05; **P < 0.01; one-way ANOVA with Tukey’s post hoc test. Bars represent the mean ± SEM. **d** Representative transmission electron microscopy images showing the accumulation of early autophagosomes in neurons in the hippocampal CA1 region. Arrowhead indicates early autophagosomes. Scale bar = 2 µm.

TLR7 reportedly participates in autophagy activation in several systems^25,26^, especially the mononuclear macrophage system^27^. The present study demonstrated that TLR7 was activated in neurons in the early stage of epileptogenesis. To test whether activation of TLR7 affected neuronal autophagy in the hippocampus, the expression profiles of autophagy-related proteins in the hippocampus were compared among four groups: WT, *Tlr7*^−/y^, WT + KA, and *Tlr*7^−/y^ + KA groups. Increased protein levels of Beclin1 and LC3BII:LC3BI, along with decreased protein levels of P62, were observed in the WT + KA group. However, the reverse changes were observed in the *Tlr7*^−/y^ + KA group. The expression of autophagy-related proteins did not differ significantly between the WT and *Tlr7*^−/y^ groups (Fig. 4a). Immunofluorescence staining revealed that staining for LC3B and NeuN double-positive neurons was significantly increased in the WT + KA group compared to that in the WT group. This phenomenon was reversed in the *Tlr7*^−/y^ + KA group, and the staining intensity did not differ between the WT and *Tlr7*^−/y^ groups (Fig. 4b, c). In addition, TEM revealed an increase in the formation of early autophagosomes in neurons in the WT + KA group, which was reduced in the *Tlr7*^−/y^ + KA group (Fig. 4d). Early autophagosomes were identified by the presence of smooth, ribosome-free, double membrane-surrounded organelles such as mitochondria, vesicles, or other cytoplasmic material^28^. These results suggested that TLR7 was involved in neuronal autophagy activation during epileptogenesis.

### TLR7 regulates KIF5A expression levels via the autophagic pathway

**Fig. 5:**
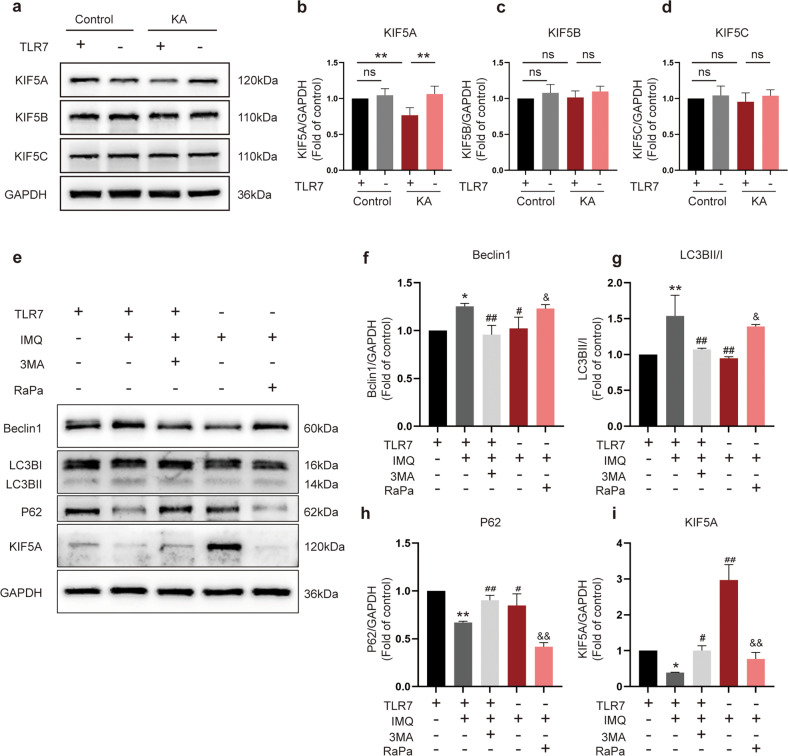
TLR7-mediated autophagy modulation of the expression of KIF5A. **a**–**d** Representative images of Kinesin family member 5 family member (KIF5A, KIF5B, and KIF5C) expression in the hippocampus were detected by western blot (*n* = 6). Quantitative analysis was performed. ns not significant; **P < 0.01; one-way ANOVA with Tukey’s post hoc test. Bars represent the mean ± SEM. **e**–**i** Representative images of autophagy-related proteins (Beclin1, LC3B, and P62) and KIF5A expression in cultured primary neurons were detected by western blot (*n* = 3). Quantitative analysis was performed. ns, not significant; *P < 0.05; **P < 0.01 versus WT group; #, *P* < 0.05; ##, *P* < 0.01 versus WT + IMQ group; ^&^*P* < 0.05; ^&&^*P* < 0.01 versus *Tlr7*^−/−^ +IMQ group. Two-way ANOVA with Dunnett’s post hoc test. Bars represent the mean ± SEM.

TLR7 deletion has been shown to alter the expression of genes related to synaptic transmission^29^, with *Kif5* being one of the top ten downregulated genes in TLR7-activated neurons^30^. To address whether TLR7-induced autophagy was involved in modulating KIF5 expression, the expression of different KIF5 family members was investigated, including KIF5A, KIF5B, and KIF5C. KIF5A mRNA expression showed a decreasing trend in the WT + KA group compared with the WT group, but there was no statistical significance between the two groups. Although TLR7 knockout appeared to reverse the decreasing trend, it was still not statistically significant (Supplementary Fig. 2c). KIF5B mRNA expression was increased in the WT + KA group compared to that in the WT group; however, this phenomenon was not reversed in the *Tlr7*^−/y^ + KA group (Supplementary Fig. 2d). KIF5C mRNA expression was not different in these groups (Supplementary Fig. 2e). Western blot analysis demonstrated markedly decreased KIF5A protein expression in hippocampal tissues in the early stage of epileptogenesis, which was reversed by TLR7 knockout (Fig. 5a, b). Additionally, the protein expression of other KIF5 family members remained unchanged in these groups (Fig. 5a, c, d).

To further examine whether KIF5A protein expression was modulated via the TLR7-dependent autophagic pathway, the expression of KIF5A and autophagy-related proteins was explored in primary cultured neurons in the presence of IMQ, an agonist of TLR7 that mimics the activation of TLR7 in the early stage of epileptogenesis in vivo. Consistent with the in vivo results, activation of TLR7 in the WT group by IMQ-induced autophagy led to decreased expression of KIF5A. However, autophagy inhibitor 3-MA addition or TLR7 deletion suppressed IMQ-induced conversion of LC3I to LC3 II, inhibited upregulation of Beclin1, and prevented decreased P62 and KIF5A expression. This phenomenon induced by TLR7 deletion could be reversed by the autophagy agonist rapamycin (Fig. 5e–i). Additionally, TLR7 knockout in primary cultured neurons exposed to rapamycin alone also exhibited autophagy activation and KIF5A expression degradation (Supplementary Fig. 2f–j). These results indicated that TLR7-mediated autophagy regulated KIF5A protein expression.

### TLR7 modulates GABA_A_R-mediated postsynaptic neurotransmission in the hippocampus

A previous study demonstrated that KIF5A is an important modulator involved in regulating neuronal network activity by affecting GABA_A_Rs trafficking^31^. The present results indicated that TLR7 activated and induced autophagy to degrade the expression of KIF5A in the early stage of epileptogenesis. Therefore, we speculated that TLR7 was involved in impairing GABAergic synaptic transmission in the hippocampus of epileptic mice. Whole-cell patch-clamp recordings were performed to investigate the synaptic transmission and neuronal excitability in the CA1 region of hippocampal slices.

**Fig. 6:**
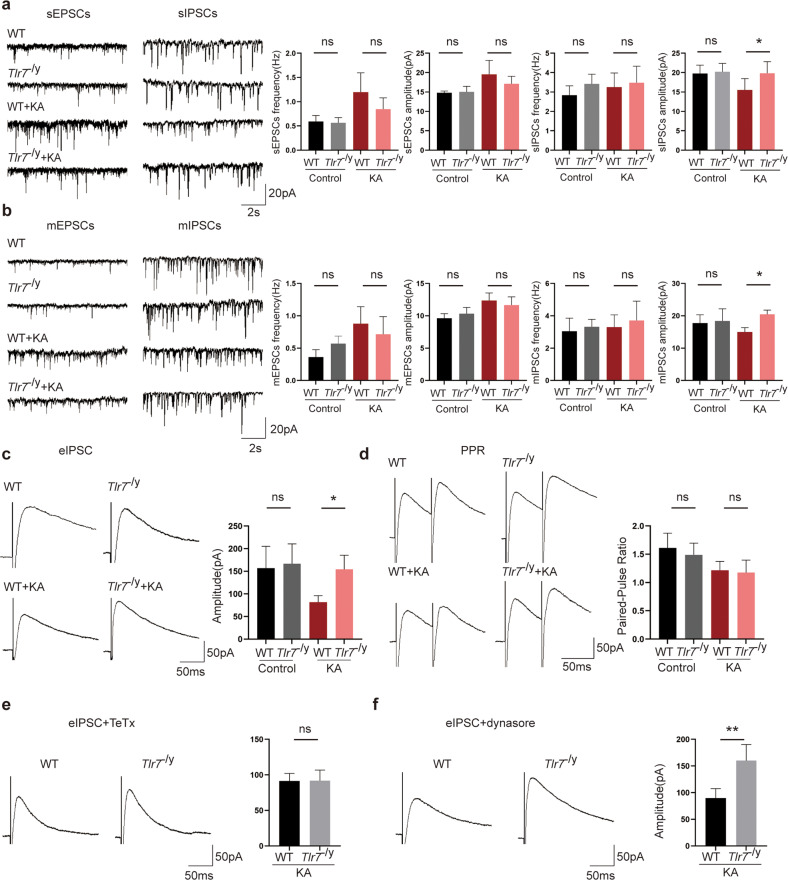
Results of patch-clamp recording. **a** Representative traces and analysis of sEPSCs and sIPSCs in the hippocampal CA1 region among the WT, *Tlr7*^−/y^, WT + KA, and *Tlr7*^−/y^ + KA groups. **b** Representative traces and analysis of mEPSCs and mIPSCs in the hippocampal CA1 region among the WT, *Tlr7*^−/y^, WT + KA, and *Tlr7*^−/y^ + KA groups. **c** Representative traces and analysis of eIPSCs in the hippocampal CA1 region among the WT, *Tlr7*^−/y^, WT + KA, and *Tlr7*^−/y^ + KA groups. **d** Representative traces and analysis of PPRs in the hippocampal CA1 region among the WT, *Tlr7*^−/y^, WT + KA, and *Tlr7*^−/y^ + KA groups. **e** Representative traces and analysis of eIPSCs between the WT + KA and *Tlr7*^−/y^ + KA groups after treatment with TeTx. **f** Representative traces and analysis of eIPSCs between the WT + KA and *Tlr7*^−/y^ + KA groups after treatment with dynasore. All above *n* = 5, ns not significant; *P < 0.05; **P < 0.01; one-way ANOVA with Tukey’s post hoc test and unpaired two-tailed Student’s *t*-test. Bars represent the mean ± SEM.

First, we addressed whether TLR7 deletion can directly affect inhibitory and/or excitatory transmission in neurons. Therefore, we examined synaptic transmission at excitatory and inhibitory synapses from hippocampal CA1 pyramidal cells in the WT, *Tlr*7^−/y^, WT + KA, and *Tlr*7^−/y^ + KA groups. Whole-cell voltage-clamp recordings revealed that neither the frequency nor the amplitude of sEPSCs was altered in CA1 pyramidal neurons (Fig. 6a). While the frequency of sIPSCs remained unaltered, the amplitude was increased in the *Tlr7*^−/y^ + KA group compared to that in the WT + KA group (Fig. 6a). sEPSCs and sIPSCs consist of AP-dependent and AP-independent events, while the latter reflects the response of postsynaptic currents to a single vesicle of the transmitter. Then, mEPSCs and mIPSCs were monitored. Neither the frequency nor the amplitude of mEPSCs was affected in these groups (Fig. 6b). However, the mean amplitude of mIPSCs in the *Tlr7*^−/y^ + KA group was increased compared to that in the WT + KA group. Additionally, the frequency of mIPSCs tended to increase in the *Tlr7*^−/y^ group but was not significantly different between the WT + KA group and the *Tlr7*^−/y^ + KA group (Fig. 6b). The frequency and amplitude of sEPSCs, sIPSCs, mEPSCs, and mIPSCs did not differ significantly between the WT and *Tlr7*^−/y^ groups (Fig. 6a, b).

An imbalance between inhibitory and excitatory synaptic transmission altered neuronal excitability. sAPs induced in pyramidal neurons were recorded to compare neuronal excitability. A lower frequency of sAPs was observed in the *Tlr7*^−/y^ + KA group than in the WT + KA group, indicating decreased neuronal excitability in these tissues. The frequency of sAPs did not change in the WT and *Tlr7*^−/y^ groups (Supplementary Fig. 3a). These data demonstrated that TLR7 deletion enhanced inhibitory synaptic transmission, resulting in decreased neuronal excitability in the early stage of epileptogenesis.

GABA_A_R-mediated inhibitory transmission provides most of the neural inhibition in the adult mammalian brain. Both extrasynaptic and synaptic GABA_A_Rs constitute GABA_A_Rs according to their neuronal localization. Extracellular GABA continuously activated extrasynaptic receptors to generate tonic currents, whereas GABA released by vesicles acted on synaptic receptors to generate phasic currents.

These two groups of GABA_A_Rs are regulated by their subunit localization^32^. To clarify the effect of TLR7 on synaptic origin, sIPSCs were recorded in the presence of low concentrations of gabazine to selectively block phasic inhibitory currents and picrotoxin to inhibit tonic currents. A remarkable increase in the amplitude of sIPSCs was observed in the *Tlr7*^−/y^ + KA group in the presence of both picrotoxin and gabazine (Supplementary Fig. 3b). These results suggested that TLR7 affected both phasic and tonic inhibition via synaptic and extrasynaptic GABA_A_Rs.

Because the amplitude of the postsynaptic current characterizes the functional efficacy of the synaptic connection, the amplitude of eIPSCs was measured. Compared to the WT + KA group, the *Tlr7*^−/y^ + KA group showed a relative increase in eIPSC amplitude (Fig. 6c). To further assess the possibility that TLR7 could influence the release of inhibitory synaptic vesicles and thus alter presynaptic release, eIPSCs were recorded in response to paired-pulse stimulation, and PPR was used to evaluate the presynaptic vesicle release probability^33^. Consistent with results showing no change in sIPSC or mIPSC frequency, no changes were found in PPRs between the WT + KA group and *Tlr7*^−/y^ + KA group. The amplitude of eIPSCs and PPRs was not altered between the WT group and *Tlr7*^−/y^ group (Fig. 6c, d). All electrophysiological data indicated that TLR7 modulated GABA_A_R-mediated inhibitory neurotransmission through a postsynaptic rather than presynaptic mechanism to impact neuronal excitability.

### TLR7 impedes GABA_A_R trafficking to the neuronal surface

**Fig. 7:**
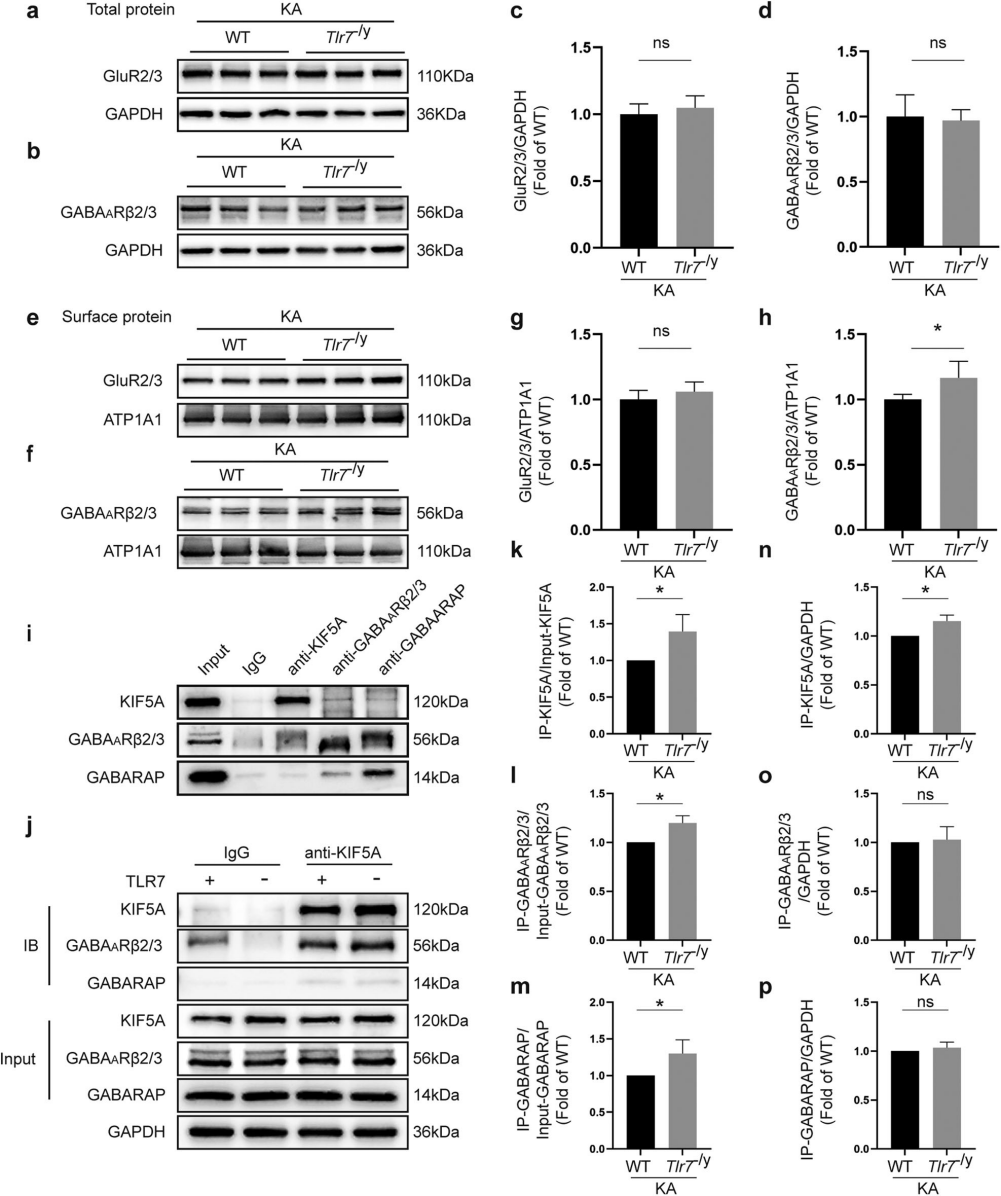
TLR7 affects the distribution of GABA_A_ receptors by disturbing the interaction among KIF5A, GABARAP, and GABA_A_Rβ2/3. **a**–**d** Representative images of total GluR2/3 and GABA_A_Rβ2/3 expression in the hippocampal tissue between the WT + KA and *Tlr7*^−/y^ + KA groups (*n* = 6). Quantitative analysis was performed. **e**–**h** Representative images of surface GluR2/3 and GABA_A_Rβ2/3 expression in the hippocampus between the WT + KA and *Tlr7*^−/y^ + KA groups (*n* = 6). Quantitative analysis was performed. ns not significant; *P < 0.05; unpaired two-tailed Student’s *t*-test. Bars represent the mean ± SEM. **i** Coimmunoprecipitation analysis demonstrated the interactions between KIF5A, GABA_A_Rβ2/3, and GABARAP in KA-injected mice. **j** Quantitative coimmunoprecipitation for detecting the influence of TLR7 on the interaction among KIF5A, GABA_A_Rβ2/3, and GABARAP in KA-injected mice. **k**–**p** Quantitative analysis for detecting the binding of KIF5A to GABARAP and GABA_A_Rβ2/3 between the WT + KA and *Tlr7*^−/y^ + KA groups (*n* = 3). ns not significant; *P < 0.05; unpaired two-tailed Student’s *t*-test. Bars represent the mean ± SEM.

To determine the mechanism by which TLR7 influenced the inhibitory postsynaptic current in the early stage of epileptogenesis, the expression of GABA_A_Rβ2/3, two key subunits of GABA_A_Rs in neurons^34,35^, was investigated. Western blot analysis demonstrated that the expression levels of total GABA_A_Rβ2/3 and glutamate receptor subtype 2 and 3 (GluR2/3) in the hippocampus did not differ in the WT, *Tlr*7 ^−/y^, WT + KA, and *Tlr*7^−/y^ + KA groups (Fig. 7a–d and Supplementary Fig. 3c–e). Similarly, surface GluR2/3 levels (Fig. 7e, g and Supplementary Fig. 3f, g) did not differ in these groups. Additionally, the surface expression of GABA_A_Rβ2/3 did not differ between the WT group and the *Tlr*7 ^−/y^ group (Supplementary Fig. 3f, h), whereas that was visibly increased in the *Tlr7*^−/y^ + KA group compared to the WT + KA group (Fig. 7f, h).

GABA_A_R accumulation on the neuronal plasma membrane is regulated via two primary mechanisms: the assembly of subunits into transport-competent GABA_A_Rs and endocytosis-mediated recycling and degradation of GABA_A_Rs^36–38^. To further distinguish the mechanism by which TLR7 upregulated surface GABA_A_R levels, eIPSCs in CA1 hippocampal neurons from the WT + KA and *Tlr7*^−/y^ + KA groups were recorded after exocytosis or endocytosis was blocked. TLR7 deletion increased the eIPSC amplitude, which was blocked by the application of the exocytosis blocker TeTx (Fig. 6e) but not by the endocytosis blocker dynasore (Fig. 6f). The results supported the idea that impaired exocytosis of GABA_A_Rs was involved in the effect of TLR7 on GABA_A_R accumulation on the neuronal plasma membrane.

To determine whether TLR7 affected the exocytosis of GABA_A_Rs, disruptions of protein–protein interactions were explored among KIF5A, GABA_A_Rβ2/3, and GABA_A_R-associated protein (GABARAP) in the hippocampus of epileptic mice using coimmunoprecipitation assays. The results showed that KIF5A coimmunoprecipitated with GABA_A_Rβ2/3 and GABARAP in the early stage of epileptogenesis (Fig. 7i), demonstrating that KIF5A interacted with GABARAP to affect the transport of GABA_A_Rβ2/3 and produce abnormal GABA_A_Rβ2/3 membrane proteins that could further influence inhibitory postsynaptic transmission in epileptic mice. TLR7 deletion increased the initial protein level of KIF5A but did not alter the initial protein levels of total GABA_A_Rβ2/3 or GABARAP in epileptic mice (Fig. 7j, n–p). TLR7 deletion appreciably increased the levels of coimmunoprecipitated KIF5A, GABA_A_Rβ2/3, and GABARAP in epileptic mice (Fig. 7j, k–m). These results confirmed that TLR7 deletion increased the initial protein expression of KIF5A, thus increasing its interaction with GABARAP and facilitating GABA_A_R trafficking to the neuronal surface in the early stage of epileptogenesis.

### Suppression caused by TLR7 depletion can be rescued by a KIF5A decrease

**Fig. 8:**
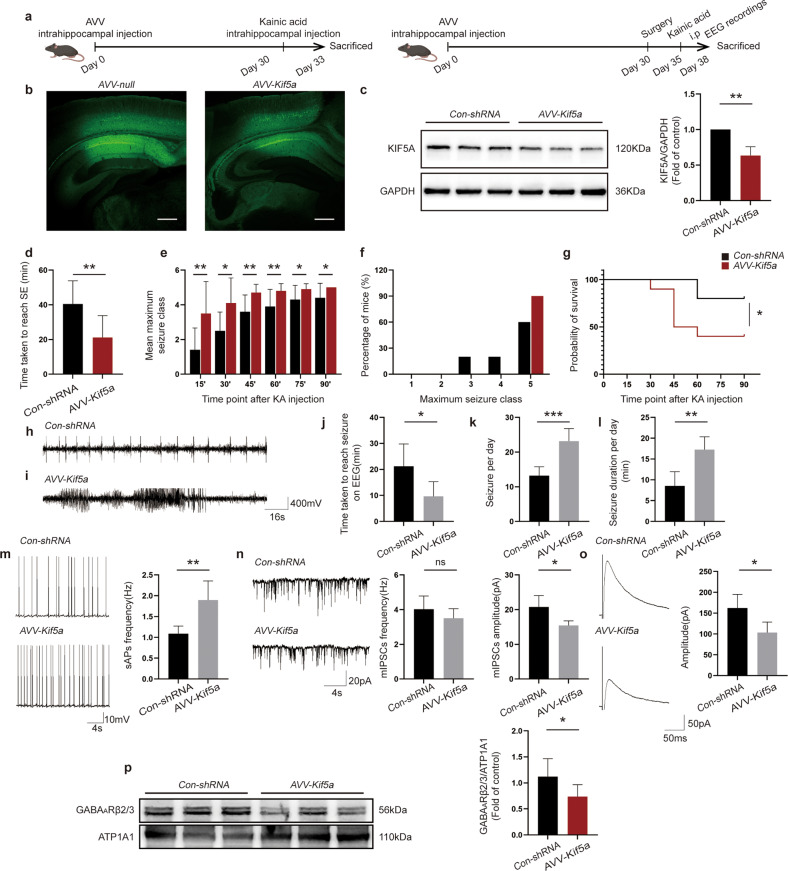
Knockdown of KIF5A in the *Tlr7*^−/y^ group rescues the effect of TLR7 deletion in the early stage of epileptogenesis. **a** Rescue experiment in *Tlr7*^−/y^ mice after intrahippocampal administration of AAV directly against *Kif5a*. **b** GFP fluorescence was detected in the mouse hippocampus in the AAV-*Kif5a* group and Con-shRNA group at Day 30 following AAV infection, Scale bars = 250 μm. **c** Hippocampal KIF5A protein expression levels were detected in KIF5A knockdown *Tlr7*^−/y^ mice 30 days after AAV injection (*n* = 5). Quantitative analysis was performed. **P < 0.01; unpaired two-tailed Student’s *t*-test. Bars represent the mean ± SEM. **d** Graph depicting the time taken to reach status epilepticus after KA intraperitoneal injection from the Con-shRNA-treated group and AAV-*Kif5a*-treated group mice (*n* = 10). **e** Graph depicting seizure progression in the Con-shRNA-treated group and AAV-*Kif5a*-treated group, illustrated as the mean maximum seizure class reached 15, 30, 45, 60, 75, and 90 min after KA intraperitoneal administration (*n* = 10). **f** Incidence of maximum seizure class reached during the course of the experiments in **e** (*n* = 10). *P < 0.05; **P < 0.01; unpaired two-tailed Student’s *t*-test. Bars represent the mean ± SEM. **g** Percentage survival between the Con-shRNA- and AAV-*Kif5a*-treated groups during the course of the experiments in **e** (*n* = 10). *P < 0.05; Mantel‒Cox log-rank test. **h** Representative EEG trace showing single spike-wave discharges in the Con-shRNA-treated group. **i** Representative EEG trace showing characteristic epileptiform spike discharges in the AAV-*Kif5a*-treated group. Latency period of seizures (**j**), the average number of seizures per day (**k**), and total duration of seizures per day (**l**) in Con-shRNA- and AAV-*Kif5a*-treated mice treated with KA (*n* = 4). **m** Representative traces and analysis of sAPs in the hippocampal CA1 region of KA-injected mice in the Con-shRNA-treated group and AAV-*Kif5a*-treated group (*n* = 5). **n** Representative traces and analysis of mIPSCs in the hippocampal CA1 region of KA-injected mice in the Con-shRNA-treated group and AAV-*Kif5a*-treated group (*n* = 5). **o** Representative traces and analysis of eIPSCs in the hippocampal CA1 region of KA-injected mice in the Con-shRNA-treated group and AAV-*Kif5a*-treated group (*n* = 5). **p** Representative images of surface GABA_A_Rβ2/3 expression in the hippocampus of KA-injected mice in the Con-shRNA-treated group and AAV-*Kif5a*-treated group (*n* = 6). Quantitative analysis was performed. ns not significant; *P < 0.05; **P < 0.01; ***P < 0.001; unpaired two-tailed Student’s *t*-test. Bars represent the mean ± SEM.

The study findings confirmed that TLR7 relied on KIF5A to modulate GABA_A_R-mediated inhibitory neurotransmission, thus participating in epileptogenesis. However, whether the knockdown of KIF5A could rescue the suppression induced by TLR7 deletion was unknown. Therefore, KIF5A rescue experiments were performed using neuron-specific AAVs carrying an shRNA directed against *Kif5a*, which was administered via intrahippocampal injection (Fig. 8a). Transfection efficiency was measured by immunofluorescence staining 30 days after AAV injection. Notably, EGFP expression was observed in the CA1 region of the injected hippocampus (Fig. 8b). Knockdown efficiency was tested by western blot analysis, which showed that KIF5A expression was decreased in the AAV-*Kif5a*-treated group compared to that in the Con-shRNA-treated group on Day 30 (Fig. 8c). These results indicated that CA1 region hippocampal neurons were successfully infected by AAV-*Kif5a*, which efficiently decreased neuronal KIF5A expression in the *Tlr7*^−/y^ group.

Next, behavioral and EEG analyses were performed between the AAV-*Kif5a-* and Con-shRNA-treated groups. Knockdown of KIF5A in the AAV-*Kif5a-*treated group rescued the prolonged latent period (Fig. 8d), slowed seizure progression (Fig. 8e), reduced maximum seizure severity (Fig. 8f), and resulted in higher mortality than that in the Con-shRNA-treated group (Fig. 8g). Single spike-wave discharges were observed in the Con-shRNA-treated group, whereas more characteristic epileptiform spike discharges were detected in the AAV-*Kif5a-*treated group (Fig. 8h, i). Additionally, the latent period was decreased, while seizure incidence and duration were increased in the AAV-*Kif5a*-treated group compared to the Con-shRNA group (Fig. 8j–l). These results indicated that the knockdown of KIF5A in the *Tlr7*^−/y^ group could rescue the suppression of seizures induced by TLR7 knockout.

Whole-cell patch-clamp recordings were performed to investigate whether the decline in KIF5A expression in the *Tlr7*^−/y^ group could rescue changes in neuronal excitatory and inhibitory synaptic transmission in hippocampal slices after intrahippocampal injection of KA. The frequency of sAPs in the AAV-*Kif5a*-treated group was significantly higher than that in the Con-shRNA-treated group (Fig. 8m). Moreover, the amplitude of mIPSCs (Fig. 8n) was markedly decreased in the AAV-*Kif5a-*treated group compared to that in the Con-shRNA-treated group. The frequency of mIPSCs did not differ between the two groups (Fig. 8n). Additionally, the amplitude of eIPSCs was also reduced in the AAV-*Kif5a-*treated group compared to that in the Con-shRNA-treated group (Fig. 8o). These results further demonstrated the important role of KIF5A in the TLR7-mediated regulation of electrophysiological phenotypes.

Finally, the surface expression levels of GABA_A_Rβ2/3 were compared between the AAV-*Kif5a*- and Con-shRNA-treated groups. The results suggested that TLR7 deletion induced an increase in surface GABA_A_Rβ2/3 expression, which was rescued by KIF5A knockdown (Fig. 8p). Taken together, these results indicated that knockdown of KIF5A expression in *Tlr7*^−/y^ mice could rescue the effects of TLR7 deletion in the early stage of epileptogenesis.

## Discussion

The importance of TLR7 in noninfectious CNS diseases is compellingly illustrated in the pathogenesis of diseases associated with neuroexcitability such as AD^5^, PD^6^, and ALS^39^. However, the mechanism underlying TLR7-mediated neuronal excitability during epileptogenesis remains unknown. Nevertheless, TLR7 expression has been shown to be significantly increased in the brain tissues of patients with tuberous sclerosis complex epilepsy^8^. The present study provided in vitro and in vivo evidence that upregulation of the TLR7-mediated autophagy pathway reduced KIF5A expression and GABA_A_R-mediated neurotransmission while inducing seizures in a murine model. First, we demonstrated that TLR7 was highly expressed in the brain during the early stage of KA-induced epileptogenesis. Immunofluorescent labeling of epileptic mouse hippocampal tissues revealed TLR7 activation, mostly in neurons and a few microglia but not astrocytes. The reason for this discrepancy with previous studies in which TLR7 was reportedly activated primarily in microglia during CNS diseases^6,40^ is that mouse hippocampal neurons are the primary nerve cells involved in epileptic pathological alterations^41^. Second, we demonstrated that TLR7 knockout prolonged the seizure-free latent period, decreased the severity of seizures, attenuated the incidence of seizures at the early and SRS stages, and alleviated abnormal discharges on EEG recordings in the early stage of epileptogenesis. Next, we verified that activating TLR7 induced neuronal autophagy to reduce KIF5A expression in the early stage of epileptogenesis, revealing that this effect was abolished after TLR7 knockout or administration of an autophagy inhibitor but rescued after treatment with an autophagy agonist. Moreover, TLR7 knockout increased GABA_A_R-mediated inhibitory synaptic transmission and suppressed neuronal excitability, partly due to the upregulation of cell surface expression of GABA_A_R via interaction among KIF5A, GABARAP, and GABA_A_Rβ2/3. Finally, exogenous manipulation to decrease KIF5A expression partially rescued the epileptic behavior and electrophysiological and molecular changes induced by TLR7 knockout both in vivo and in vitro. Based on this evidence, blocking TLR7-mediated autophagy in the early stage of epileptogenesis could provide a promising preventive approach against TLE.

Various TLRs expressed in neurons, astrocytes, and microglia are conducive to the immunological responses of the CNS^42^. Early studies exploring the mechanisms responsible for recognizing and fighting infection were critical to the discovery of the immune functions of TLRs^43^. Recently, TLR7 has been reported as a prominent mediator of noninfectious CNS diseases. However, the present study is the first to investigate the biological mechanism of TLR7 in a mouse model of TLE. We provide compelling evidence that TLR7 is highly expressed primarily in the early stage of epileptogenesis after brain insult but before the SRS stage. Our results are consistent with those of previous studies in which FL-TLR7 (~120 kDa) in mouse macrophages and dendritic cells was degraded by asparagine endopeptidase and cathepsin to generate a TLR7 C-terminal fragment (~60 kDa), which was sufficient and necessary for TLR7 activity^44,45^. In the present study, we demonstrate for the first time that this process was also observed in the mouse brain, which aligns with a previous study reporting that FL-TLR9 and C-terminal TLR9 were activated in midbrain dopamine neurons and participated in PD pathology^46^.

Autophagy is a double-edged sword in many diseases, including diabetic retinopathy, ischemic stroke, and epilepsy^11,12,15^. Basal autophagy has been shown to promote cell survival against stressful circumstances by generating energy through the reuse of intracellular components and controlling the clearance of other components. When stress is excessive and beyond the maximum adaptive ability of the cell, autophagy induces cell death. The degree and duration of autophagy activation together determine whether autophagy is beneficial or harmful^12,47^. Accumulating evidence suggests that abnormal autophagy contributes to neuronal damage in the early stage of epileptogenesis after KA-induced SE^48,49^. In addition to neuronal damage, the impaired synaptic function is also attributed to autophagy. Synaptic dysfunction is an inevitable consequence of disordered autophagy in neurons^50^, affecting synaptic development, neurotransmitter release, postsynaptic function, and synaptic plasticity^51,52^. Notably, TLR7 detects the presence of microbial invaders by recognizing PAMPs, whose ligands, single-stranded RNAs, and agonists are potent inducers of autophagy^53^. Indeed, TLR7 was shown to activate B-cell autophagy and induce systemic lupus erythematosus by delivering RNA ligands to endosomes, where this innate immune receptor resides^54^. Moreover, selective autophagy was induced by stimulating TLR7 with IMQ to control *Mycobacterium tuberculosis* growth in mouse macrophages^27^. These studies suggested a relationship between TLR7 expression and autophagy. Our results indicated that TLR7-dependent neuronal autophagy is responsible for accelerating epileptogenesis.

KIF5 is encoded by three distinct genes, *KIF5A*, *KIF5B*, and *KIF5C*, comprising a superfamily of microtubule-dependent motors that play important roles in organelle transport and cell division^55^. We reported that KIF5A protein expression but not mRNA expression was significantly reduced by activating TLR7 in the KA group, and this trend was reversed by TLR7 knockout. TLR7 is located in cytoplasmic vesicles, the endoplasmic reticulum, and lysosomes. Lysosomes play an indispensable role in different types of autophagy. Autophagic degradation of proteins and organelles has been shown to play critical roles in neurotransmission^56,57^. Studies have reported that autophagic degradation of GABA_A_Rs, alpha-amino-3-hydroxy-5-methyl-4-isoxazolepropionic acid (AMPA) receptor, and glutamate receptor subtype 1 (GluR1) controls synaptic transmission via postsynaptic mechanisms^58,59^. However, the mechanism by which autophagy contributes to the degradation of postsynaptic receptor proteins remains unknown. Moreover, the mechanisms by which neuronal autophagy regulates synaptic transmission are elusive. This study provides insight into the unknown mechanism of activated TLR7-induced autophagic degradation of KIF5A, which is a key regulator of GABA_A_R-mediated inhibitory synaptic transmission and may represent a new therapeutic target for epileptogenesis.

Glutamate and GABA are the principal neurotransmitters that play critical roles in the imbalance between neuronal excitation and inhibition, which may contribute to epileptogenesis^32^. Neuronal excitability can be altered by increased levels of excitatory neurotransmitters and/or decreased levels of inhibitory neurotransmitters. GABA is considered to be the major inhibitory neurotransmitter in the brain, and GABA disorders have been implicated in epileptogenesis. GABA interacts with type A, B, and C receptors, among which GABA_A_Rs provide the majority of the inhibitory currents in neurons. Abnormal levels of GAB_A_Rs in the synaptic cleft can lead to epileptogenesis^60^. In the present study, KIF5A, an essential regulator of GABA_A_R transport^31^, was degraded by TLR7-dependent autophagy in the early stage of epileptogenesis. Furthermore, our results indicated that TLR7 disturbed the interaction among KIF5A, GABA_A_Rβ2/3, and GABARAP to decrease the membrane expression levels of GABA_A_Rβ2/3, hinting at the possibility that regulation by TLR7 of GABA_A_R-mediated inhibitory synaptic transmission involved the regulation of GABA_A_R surface expression.

This study had some limitations that will be the focus of future research. First, while the activation of TLR7 in neurons was mainly responsible for mediating neuronal excitability-induced epileptogenesis, the contributions of microglial mechanisms were not elucidated in the TLE model, as microglia were rarely activated. Whether TLR7 activation in microglia plays a regulatory role in neuronal excitability warrants further study. Second, the specific mechanisms by which autophagy degrades synaptic transport-related or synaptic receptor proteins require more research. Third, autophagic degradation of KIF5A may affect KIF5A-dependent axonal transport deficiency, causing autophagic flux impairment via disturbance of lysosomal function^61^, thus driving epileptogenesis. This hypothesis will be explored in future experiments. Last, in this research, we used C57BL/6 mice as the control group, even though this mouse strain was recommended on Jackson’s website, but littermates could be a more rational choice for the control group. Thus, we considered it to be a limitation of this study.

In conclusion, this study demonstrated that TLR7 activation promoted epileptogenesis in a murine model via modulation of autophagic degradation of KIF5A, interactions among KIF5A, GABA_A_Rβ2/3, and GABARAP, and neuronal surface expression of GABA_A_Rβ2/3, which together contributed to decreased GABA_A_R-mediated inhibitory synaptic transmission. The study findings demonstrated a novel mechanism underlying the harmful effect of autophagy in the early stage of epileptogenesis and provided further evidence that TLRs participate in noninfectious CNS diseases.

## Supplementary information

supplement materials
