# Supplementary material for: Activation of TLR7-mediated autophagy increases epileptic susceptibility via reduced KIF5A-dependent GABAA receptor transport in a murine model
Source: Exp Mol Med. 2023 Jun 1;55(6):1159–73. doi: 10.1038/s12276-023-01000-5 (PMC10317981; doi:10.1038/s12276-023-01000-5)
Supplement: Supplementary file 1 — supplement materials [file 12276_2023_1000_MOESM1_ESM.pdf]

Activation of TLR7-mediated autophagy increases epileptic susceptibility via reduced KIF5A-dependent $GABA_A$ receptor transport in a murine model

Jing Liu[ab], Pingyang Ke[a], Haokun Guo[a], Juan Gu[a], Yan Liu[a], Xin Tian[a], Xuefeng Wang [a*] and Fei Xiao[ac*]

This PDF file includes:

Supplementary Methods details

Supplementary Methods Table 1

Supplementary Figures and Figure legends

Supplementary Methods details

Racine scale

(1) catatonic posture followed by facial clonus; (2) masticatory movements, wet-dog shakes and head nodding; (3) continuous body tremor or wet-dog shakes; (4) unilateral or bilateral forelimb clonus; (5) repeated rearing and falling.

Surgeries for electrode implantations

Mice were anesthetized by intraperitoneal injection of pentobarbital (100mg/kg), then mounted on a stereotaxic apparatus and prepared the scalp for incision. An incision was made along the scalp midline and the 8201 mouse head mount was affixed to the skull with cyanoacrylate glue. The front edge of the head mount should be placed 3-3.5 mm anterior of bregma. A correctly sized pilot hole was opened in the skull by twisting a 23-gauge needle and the electrode was screwed into place. The epoxy was applied between the screw head and the silver coating on the holes of the head mount. A small pocket was made in the nuchal muscles using a pair of forceps to insert the EMG wire into the opening and straighten the wire. Incisions were closed with dental cement to insulate and protect the EEG leads.

EEG Data Acquisition and analysis

Seizures in EEG were recognized on characteristic spike-wave EEG discharges. Data were analyzed by a blinded researcher using Sirenia Seizure software (Pinnacle Technology). EEG signals were filtered using a 10 Hz high pass filter, and seizure events were detected by blinded manual scoring. Seizures were defined as high-frequency, high-voltage, synchronised, heterogeneous spike-wave discharges with amplitudes at least 2-fold greater than the background, lasting more than 6 s in duration.

Protein extraction and western blot analysis

The hippocampal and cortex tissues were isolated from the mice brain on 3 days after intrahippocampal injection of KA and the cells were isolated from C57BL/6 newborn mice (P0-P1). Each hippocampal tissue was processed in duplicate. The cells or tissue samples were RIPA protein extraction kit (Beyotime Biotechnology China) containing phenylmethylsulfonyl fluoride (PMSF; Beyotime Biotechnology China). After incubation on ice for 30 min for the completed lysis and centrifugated at 15,000 x g for 20 min. Minute™ Plasma Membrane Protein Isolation and Cell Fractionation Kit (Invent, SM-005) was used for membrane protein extraction. An enhanced bicinchoninic acid (BCA) protein assay kit (Beyotime Institute of Biotechnology) was used to detect protein concentrations. SD-SPAGE Sample Loading Buffer-5× (Beyotime Institute of Biotechnology) was used to denature the proteins. Protein samples were separated by SDS-PAGE (7.5% spacer gel, 10% separating gel and 12.5% separating gel) (Epizyme Biotechnology) and electrotransferred onto 0.45- or 0.2μm polyvinylidene difluoride membranes (Millipore, Billerica, MA, USA). The membranes were blocked with 5% nonfat dry milk in tris-buffered saline with Tween (TBST) at RT for 1 hour and then incubated with primary antibodies overnight at 4°C. Then horseradish peroxidase (HRP)-conjugated anti-rabbit secondary antibodies. The bands were visualized using Western Bright ECL (Advansta, US) and a Fusion FX5 image analysis system (Vilber Lourmat, France).

Immunofluorescence analysis

The brain sections were blocked with 10% goat serum (Boster Bio, Wuhan, China) or 5% donkey serum (Sigma-Aldrich), and 0.4% Triton X-100 in PBS for 1 h. The sections were then incubated overnight with mixed primary antibodies in a humidified chamber at 4 °C, washed with 1×PBS to remove unbound primary antibodies, and incubated with secondary antibodies in the dark at RT for 1 h.

Images were captured using a laser-scanning confocal microscope (Nikon, Tokyo, Japan) under an IX 70 inverted microscope (Olympus, Tokyo, Japan) equipped with a FluoView FVX confocal scan head.

Transmission electron microscopy (TEM)

The brain tissue samples were fixed with 1% osmium tetroxide for 1 h and dehydrated using a graded ethanol series (50, 70, 80, 90, and 95%) for 15 min. The tissues were then placed in epoxy resin and polymerised at 60 °C for 2 h. Finally, ultrathin sections (60 nm) were acquired, which were double stained with uranyl acetate and lead citrate.

Hippocampal neuronal culture

First, brains were removed from mice and placed in 60-mm dishes with dissection medium on ice. Hippocampus were dissected form brain and digested with 0.25% trypsin solution (Invitrogen) dissociation for 15 min at 37°C with gentle shaking every 5 min. Dissociated hippocampal neurons were cultured in poly-L-lysine (Sigma-Aldrich)-coated six-well dishes with DMEM/F-12 medium (Gibco) containing 10% FBS, 10% fetal bovine sera (Gibco) and 1% (v:v) penicillin/streptomycin in a 5% CO2 humidified atmosphere at 37°C. At 4 h after seeding, the medium was replaced by Neurobasal-A Medium (Gibco) supplemented with 2% B-27 (Gibco) and 1% GlutaMAX (Gibco) for 14 d. During this period, the medium was changed every 2 to 3 days to ensure adequate nutrition of the cells.

Whole-cell patch-clamp recordings

Cutting solution containing (in mM): 60 NaCl, 100 sucrose, 2.5 KCl, 1.25 NaH_2PO_4•$2H_2O$, 20 D-glucose, 26 $NaHCO_3$, 1 $CaCl_2$, and 5 $MgCl_2$ •$6H_2O$ (pH 7.4, 290-300 mOsm/L), saturated with 5% CO_2 and 95% O_2.

Artificial cerebrospinal fluid containing (in mM): 125 NaCl, 3 KCl, 1.25 NaH2 PO4•2H2O, 1.3 MgCl2,15 D-glucose, 26 NaHCO3, and 2 CaCl2 (pH 7.4, 290-300 mOsm), saturated with 5% CO_2 and 95% O_2.

For sAPs recording, the glass pipette solution contained (in mM):17.5 KCl, 0.5 EGTA, 122.5 K-gluconate, 10 HEPES, and 4 ATP, pH adjusted to 7.2 with KOH.

For sEPSCs recording, the glass pipette solution contained (in mM): 130 $CsMeSO_4$, 10 $CsCl_2$, 10 HEPES, 4 NaCl, 1 $MgCl_2$, 1 EGTA, 5 MgATP, 0.5 Na_3GTP, 12 hosphocreatine, and 5 N-methyl-D-glucamine (NMG) (pH 7.4, 280-290 mOsm). sEPSCs were recorded in the presence of 100 μM picrotoxin (PTX), a natural noncompetitive antagonist of GAB_AA receptor.

For sIPSC recording, the glass pipette solution contained (in mM): 100 CsCl, 10 HEPES, 1 $MgCl_2$, 1 EGTA, 5 MgATP, 0.5 Na3GTP, 12 phosphocreatine, 30 NMG ((pH 7.4, 280-290 mOsm). sIPSCs were recorded in the presence of 20 μM 6,7-dinitroquinoxaline-2,3(1H,4H)- dione (DNQX) and 50 μM (2R)-amino-5-phosphonovaleric acid (D-APV),that blocked excitatory transmission mediated by AMPA/kainate and N-methyl-D-aspartate (NMDA) receptors, respectively.

For mEPSCs and mIPSCs recording, the same method was used to record sIPSCs and sEPSCs above. The only difference was that 1μM TTX was added to ACSF totally preventing the occurrence of synaptic events caused by spontaneous discharges of pyramidal cells. sIPSCs were recorded in the present of gabazine (0.5 μM) to selectively block the phasic inhibitory currents or picrotoxin (10 μM) to inhibit the tonic currents. All reagents were purchased from Sigma-Aldrich, except gabazine and tetanus toxin (TeTx), which were purchased from MedChemExpress.

Supplementary Table 1. Primer sequences

ID	Gene	Primer sequence	
170743	*Tlr7*	F (5`-3`)	TGATCCTGGCCTATCTCTGAC
		R (5`-3`)	CGTGTCCACATCGAAAACAC
16572	*Kif5a*	F (5`-3`)	AGAACGAGAAGAGCGCCAG
		R (5`-3`)	GAACGAACAGCTTGCGAAGG
16573	*Kif5b*	F (5`-3`)	TCGTGTGTTCCAGTCAAGCA
		R (5`-3`)	CTCCATCGTGTGGGTCTTCC
16574	*Kif5c*	F (5`-3`)	TGGCACCAACGATGTGAAGA
		R (5`-3`)	CTGCGGTTCACGAGAGACTT
14433	*Gapdh*	F (5`-3`)	TTGTCATGGGAGTGAACGAGA
		R (5`-3`)	CAGGCAGTTGGTGGTACAGG

Supplementary Figures and Figure legends

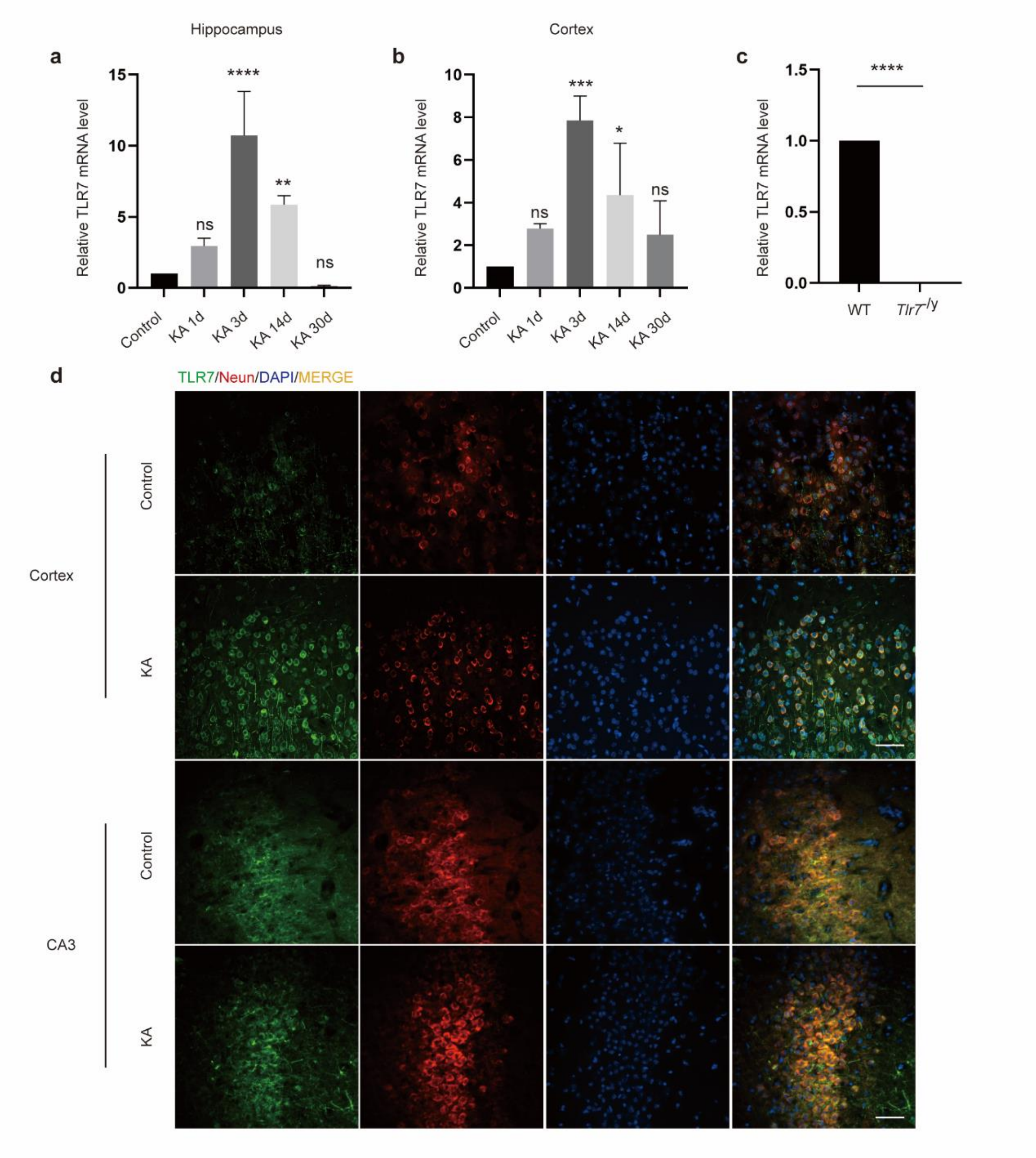

Supplementary Fig 1. Quantitative real-time PCR analysis of the expression of TLR7 mRNA from hippocampus (a) and cortex (b) in different time points after injection of KA (n=3). c. TLR7 mRNA levels in *Tlr7*$^{-/y}$ mice brain (n=3). ns, not significant; *, P < 0.05; **, P < 0.01; ***, P < 0.001; ****, P < 0.0001; one-way ANOVA with the Tukey's post-hoc test. Bars represent mean±SEM. d. Double immunofluorescence staining showed TLR7 was activated in KA and co-localized with NeuN in cortex and CA3 region of hippocampus. Scale bar = 50 µm.

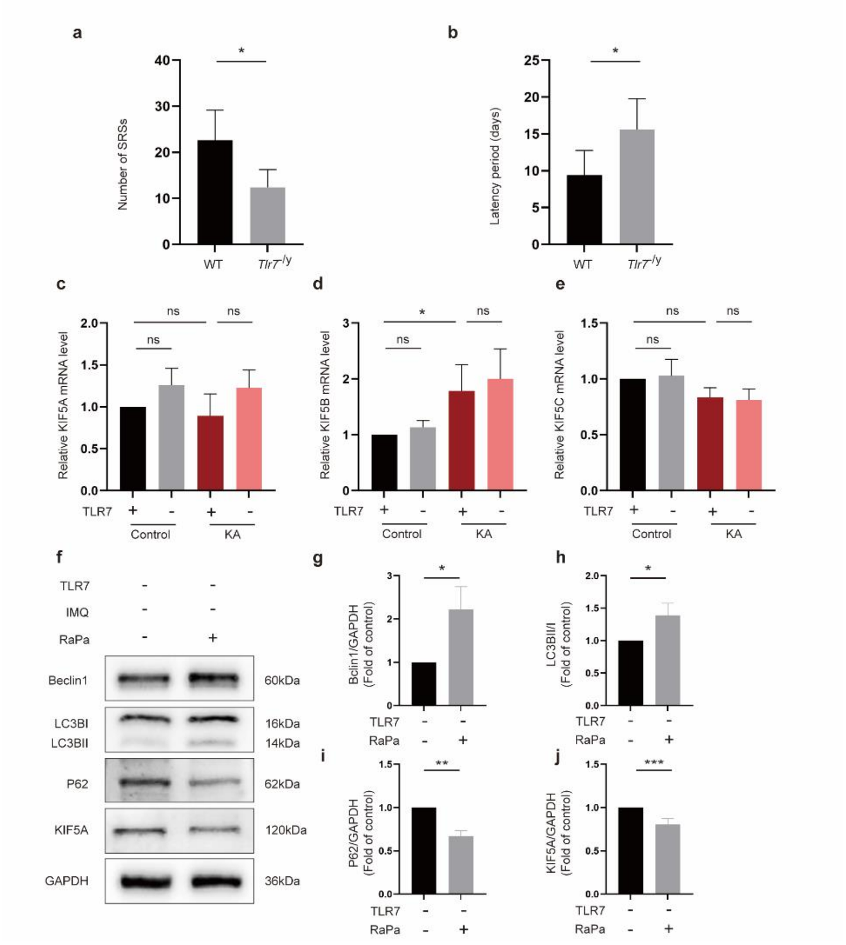

Supplementary Fig 2. a and b. Quantitative analysis of the total number of SRSs and latency of SRSs between WT+KA and *Tlr7*$^{-/y}$+KA groups (n=5). *, P < 0.05; unpaired two-tailed Student't test. Bars represent mean ± SEM. c-e. Quantitative real-time PCR analysis of the expression of Kinesin family member 5 family members (KIF5A, KIF5B, KIF5C) in the hippocampus were detected in different groups (n=5). ns, not significant; *, P < 0.05; one-way ANOVA with the Tukey's post-hoc test. Bars represent mean ± SEM. f-j. Representative images of autophagy related proteins (Beclin1, LC3B and P62) and KIF5A expression in cultured primary neuron were detected by Western blot (n=3). Quantitative analysis was performed. *, P < 0.05; **, P < 0.01; ***, P < 0.001; unpaired two-tailed Student't test. Bars represent mean ± SEM.

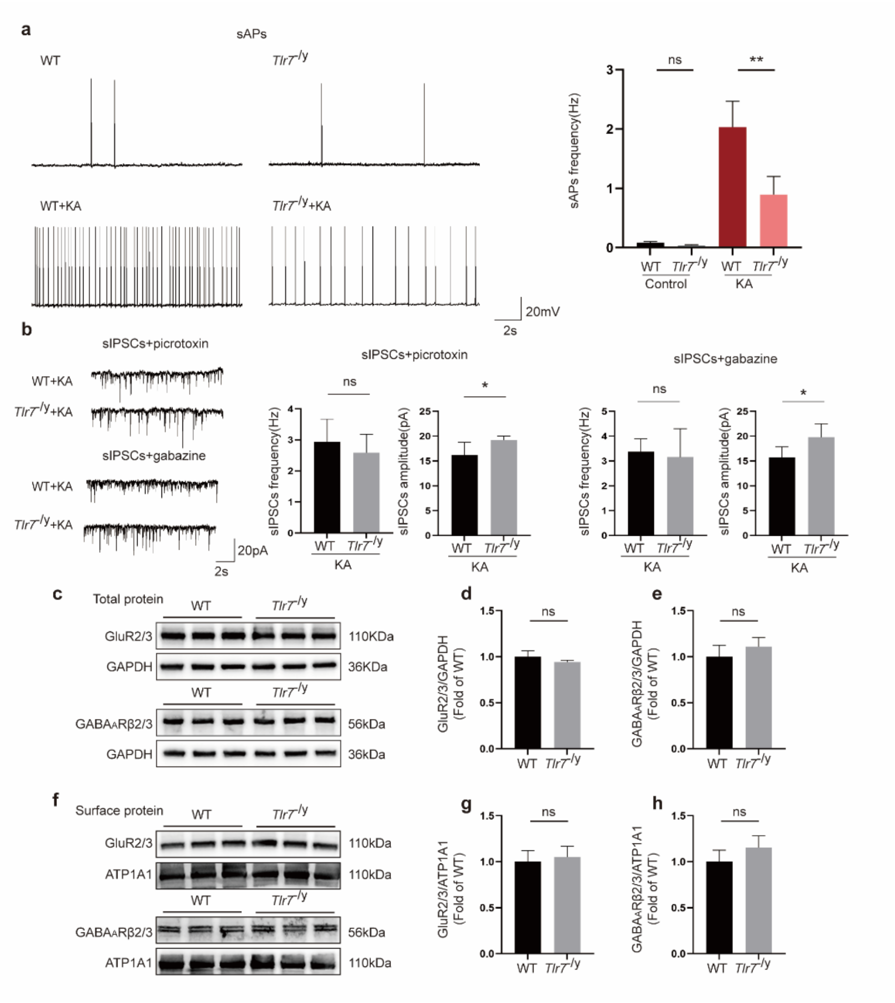

Supplementary Fig 3. a. Representative traces and analysis of sAPs in the hippocampal CA1 region among WT, *Tlr7*$^{-/y}$, WT+KA, *Tlr7*$^{-/y}$+KA groups. ns, not significant; **, P < 0.01; one-way ANOVA with the Tukey's post-hoc test. Bars represent mean ± SEM. b. Representative traces and analysis of sIPSCs between WT+KA and *Tlr7*$^{-/y}$+KA groups after treatment with picrotoxin or gabazine. c-e. Representative images of total GluR2/3 and GABA$_A$Rβ2/3 expression in the hippocampal tissue between WT and *Tlr7*$^{-/y}$ groups (n=6). Quantitative analysis was performed. f-h. Representative images of surface GluR2/3 and GABA$_A$Rβ2/3 expression in the hippocampus between WT and *Tlr7*$^{-/y}$ groups (n=6). Quantitative analysis was performed. ns, not significant; *, P<0.05; unpaired two-tailed Student't test. Bars represent mean ± SEM.
